# Supplementary material for: CB1R regulates soluble leptin receptor levels via CHOP, contributing to hepatic leptin resistance
Source: eLife. 2020 Nov 19;9:e60771. doi: 10.7554/eLife.60771 (PMC7728447; doi:10.7554/eLife.60771)
Supplement: Supplementary file 1. [file elife-60771-supp1.docx]

**Supplementary Table 1. mRNA Primers**

| Gene  (*mus musculus)* | Forward | Reverse |
| --- | --- | --- |
| *Lepr-s (Ob-Re)* | TAATGAAGATGATGGAATGAAG | ATTGCCAGTCTACAGTGTCA |
| *Ddit3* (*Chop*) | CTGGAAGCCTGGTATGAGGAT | CAGGGTCAAGAGTAGTGAAGGT |
| *Ppp1r15a* (*Gadd34*) | TGAGTACAATGCAAAGCCAGGA | CCCTCTTCTCTAGCCACCACCT |
| *Atf4* | CCTGAACAGCGAAGTGTTGG | TGGAGAACCCATGAGGTTTCAA |
| *Ubc* | GCCCAGTGTTACCACCAAGA | CCCATCACACCCAAGAACA |
| *Adam10* | TGGAGTAGAGGAAGGAGCCC | CTTTCAGCCAGAGTTGTGCG |
| *Adam17* | GTGCTGGGAAGATCACCTCC | CACCACCTCTCTGGGAAACC |

**Supplementary Table 2. ChIP primers**

| Gene  (*mus musculus)* | Forward | Reverse |
| --- | --- | --- |
| *Gapdh* | ACCAGGGAGGGCTGCAGTCC | TCAGTTCGGAGCCCACACGC |
| *Ppp1r15a* (*Gadd34*) | GTTGGCGCAGATTGAGTCAG | GGTTCATGTCGCCCTCAG |
| *Lepr* promoter | ATCTAAGGCACTGGGTGTC | TCTGGGTCCGGAGCGCTG |
